# Development of Eco-friendly Soy Protein Isolate Films with High Mechanical Properties through HNTs, PVA, and PTGE Synergism Effect

**DOI:** 10.1038/srep44289

**Published:** 2017-03-10

**Authors:** Xiaorong Liu, Ruyuan Song, Wei Zhang, Chusheng Qi, Shifeng Zhang, Jianzhang Li

**Affiliations:** 1 MOE Key Laboratory of Wooden Material Science and Application, Beijing Key Laboratory of Wood Science and Engineering, College of Material Science and Technology, Beijing Forestry University, Beijing, 100083, China

## Abstract

This study was to develop novel soy protein isolate-based films for packaging using halloysite nanotubes (HNTs), poly-vinyl alcohol (PVA), and 1,2,3-propanetriol-diglycidyl-ether (PTGE). The structural, crystallinity, opacity, micromorphology, and thermal stability of the resultant SPI/HNTs/PVA/PTGE film were analyzed by the Attenuated total reflectance-Fourier transformed infrared (ATR-FTIR) spectroscopy, X-ray diffraction (XRD), UV-Vis spectrophotometry, scanning electron microscopy (SEM), and thermo-gravimetric analysis (TGA). The SPI/HNTs/PVA/PTGE film illustrated that HNTs were uniformly dispersed in the SPI matrix and the thermal stability of the film was enhanced. Furthermore, the tensile strength (TS) of the SPI/HNTs/PVA/PTGE film was increased by 329.3% and the elongation at the break (EB) remained unchanged. The water absorption (WA) and the moisture content (MC) were decreased by 5.1% and 10.4%, respectively, compared to the unmodified film. The results highlighted the synergistic effects of SPI, HNTs, PVA, and PTGE on the mechanical properties, water resistance, and thermal stability of SPI films, which showed excellent strength and flexibility. In short, SPI films prepared from HNTs, PVA, and PTGE showed considerable potential as packaging materials.

There has recently been a growing demand for fully sustainable and eco-friendly materials due to the general overdependence on petrochemical-based synthetic polymers^1^. Many efforts have been conducted to fabricate innovative packaging materials with natural origin (e.g., proteins, fats, and carbohydrates)^2^,^3^,^4^. Soy protein isolate (SPI) shows notable potential as a packaging material due to its inherent advantages of renewability, biocompatibility, biodegradability, and film-forming capacity^5^. The inferior mechanical properties and high moisture sensitivity of the developed SPI-based films have limited their practical application, however.

The use of epoxide compounds has been also explored. The results that an epoxide compound such as 1,2,3-propanetriol-diglycidyl-ether (PTGE) or epoxidized soybean oil (EPO) can significantly enhance the mechanical properties of SPI-based composites^5^. The tensile strength (TS) of the resultant film crosslinked by PTGE and EPO were increased by 197% and 139%, respectively, compared to that of the control group. Unfortunately, the elongation at break (EB) was greatly reduced by 67% and 70%, respectively, due to the crosslinkage effect. The current challenge is to increase the TS provided EB is not compromised.

By combining the advantages of many components, nanocomposites are attracting growing interest in this field as well. They are fabricated by incorporating a suitable nanophase into the polymer matrix thereby enhancing features like mechanical properties and thermal stability^6^. The incorporation of organic/inorganic nanofillers (e.g., starch nanocrystals, cellulose nanocrystals, nano-TiO_2_) retained the EB by partly scattering the stress of the crosslinking system^7^,^8^,^9^.

As the nano-strengthening phase, clay nanotubes are interesting prospective fillers for polymeric composites due to their large surface area, high length-to-diameter (L/D) ratio, good physicochemical properties, and low cost^10^,^11^,^12^. In particular, the two-layered aluminosilicate clay halloysite nanotubes (HNTs) with a length of 0.2–2 mm, outer diameter of 40–70 nm, and inner diameter of 10–30 nm, are promising candidates as catalyst support and reinforcing fillers owing to their inherently hollow nanotube structure, different outer and inner chemistry, and cost effectiveness^13^,^14^,^15^,^16^. For example, the thermal stability of polypropylene/HNTs nanocomposites is significantly increased after the incorporation of HNTs^17^. HNTs can also be used as functional fillers to enhance the mechanical properties of nanocomposites for industrial application and coating materials^18^,^19^.

However, aggregation in the nanocomposite preparation stage results from the strong inter-particle interactions of high filler content HNTs limits their homogeneous dispersion and is proved harmful to the mechanical properties of the resultant film^20^,^21^. Chemical surface modifications, including coupling agent modification, silylation, and intercalating modification, have been tested in the past in effort to promote the dispersion of HNTs and increase their compatibility with the matrices^22^,^23^,^24^,^25^,^26^. Unfortunately, these approaches are difficult to be carried out and affect the reinforcing performance of HNTs. Poly-vinyl alcohol (PVA), a non-toxic and biodegradable synthetic polymer, may effectively enhance the uniform dispersion of HNTs in the SPI matrix^27^.

In this study, the novel nanocomposite films that combined the advantages of SPI, HNTs, PVA, and PTGE were prepared via the casting method and the mechanical properties, water resistance, chemical structures, micromorphology, opacity, crystallinity, and thermal stability of the resultant films were thoroughly investigated.

## Results and Discussion

### Structural Analysis of SPI-based Films

**Figure 1:**
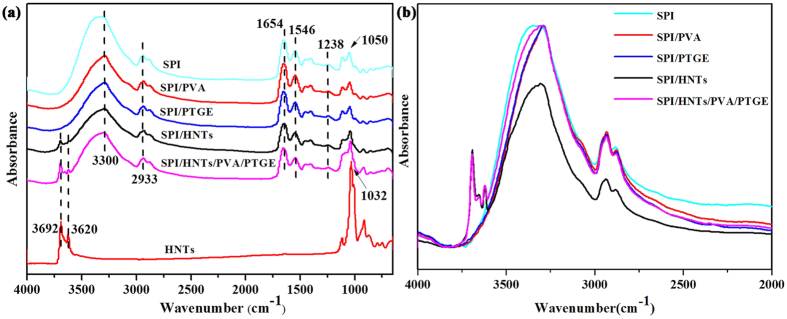
ATR-FTIR spectra of the individual HNTs and SPI-based films: SPI, SPI/PVA, SPI/PTGE, SPI/HNTs, and SPI/HNTs/PVA/PTGE.

The ATR-FTIR spectra of HNTs and different SPI-based films are shown in Fig. 1a. In the case of HNTs, two absorption peaks at 3692 and 3620 cm^−1^ could be attributed to O-H bending (which connects the two Al atoms) along with other bands characteristic of the inorganic alumino-silicate structure of HNTs^24^. The spectrum showed absorption bands around at 1032 and 914 cm^−1^ attributed to the Si-O stretching vibrations and Al-OH vibration bands, respectively^28^. All SPI films exhibited characteristic peaks of amide I (C-O stretching), amide II (N-H bending), and amide III (C-N and N-H stretching) at 1654, 1546, and 1238 cm^−1^, respectively^29^. The peak at 2933 cm^−1^ was assigned to the −CH_2_ groups stretching vibrations and the peak situated at 1032 cm^−1^ in all spectra might be related to C–O stretching, owing to the glycerol^30^,^31^,^32^. The position of peaks at 3310 cm^−1^ in the SPI film moved to 3288 cm^−1^, indicating the physicochemical cross-linking interactions between SPI and PTGE. Furthermore, the amplification spectrum (4000 cm^−1^ to 2000 cm^−1^) is shown in Fig. 1b, the intensities of peaks at 3310 cm^−1^ in SPI/HNTs film decreased considerably, indicating that the modification was accompanied by the consumption of surface hydroxyl groups of HNTs. Compared with original HNTs, the characteristic peaks at 3692, 3620, and 1032 cm^−1^ of the HNTs nanoparticles remained in the SPI/HNTs and SPI/HNTs/PVA/PTGE films, suggesting that the structure of HNTs was not changed in resultant films^33^.

### The XRD spectra of SPI-based films

**Figure 2:**
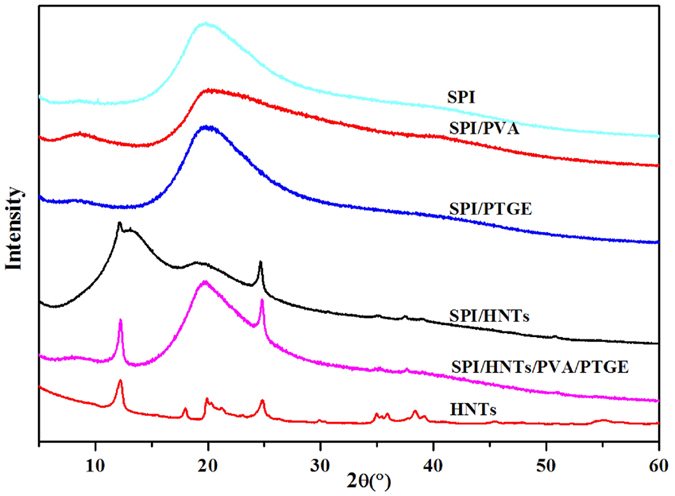
XRD pattern of the individual HNT and SPI-based films: SPI, SPI/PVA, SPI/PTGE, SPI/HNTs, and SPI/HNTs/PVA/PTGE.

The XRD spectra of HNTs and SPI-based films are presented in Fig. 2. For HNTs, three characteristic XRD peaks were detected at 2θ = 12.2°, 19.9° and 25°, which are corresponding to the reflection planes of (001), (020), (110) and (002), respectively^21^,^34^,^35^. The diffraction peaks of unmodified SPI film at 8.6°and 19.8° corresponded to α-helix and β-sheet structures of the SPI secondary structure, respectively. These findings were in agreement with our previous results^5^. The characteristic diffraction peak positions of SPI did not change after PVA was introduced^36^. With the incorporation of PTGE, the diffraction peaks of SPI at 8.6° shifted to 8.0° in the film, indicating that the lattice constant in the cubic cell increased and the crystal lattice of the SPI α-helix changed^5^. As the XRD pattern of the SPI/HNTs/PVA/PTGE film shown, three additional peaks at around 2θ = 12.2°, 19.9° and 25° were observed, corresponding to the crystalline structure of HNTs. This result indicated that HNTs was uniformly dispersed in the SPI matrix and the crystal structure of HNTs was not altered^26^,^37^.

The calculated degrees of crystallinity of HNTs and the SPI, SPI/PVA, SPI/PTGE, SPI/HNTs, SPI/HNTs/PVA/PTGE films are listed in Table 1. The crystallinity of SPI/HNTs and SPI/HNTs/PVA/PTGE films increased from 23.1% to 34.4% and 23.1% to 36.3%, respectively, mostly due to the crystal structure of HNTs. The significant increase in crystallinity in the SPI/HNTs films might be caused by the strong hydrogen bond interactions between HNTs and SPI^38^. The SPI/HNTs/PVA/PTGE film was attributed to the synergistic effects of SPI, HNTs, PVA, and PTGE. These conclusions were consistent with the SEM, and TGA results.

### Opacity Analysis of SPI-based Films

**Figure 3:**
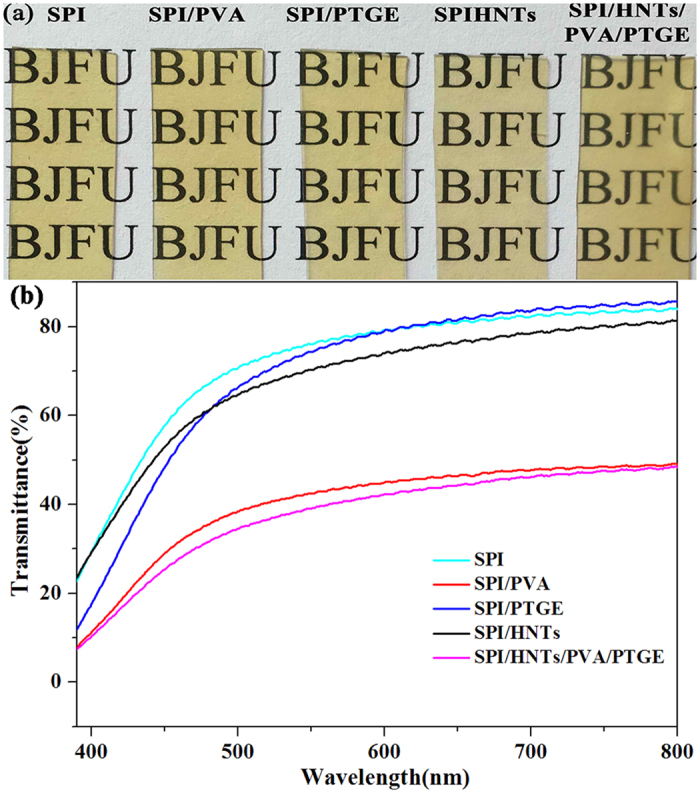
Appearance (a) and UV/Vis spectra (b) of SPI-based films: SPI, SPI/PVA, SPI/PTGE, SPI/HNTs, and SPI/HNTs/PVA/PTGE.

The appearances of SPI-based films are shown in Fig. 3a. The films became transparent when modifiers, such as PVA, PTGE and HNTs alone or together, were incorporated. The incorporation of HNTs did not affect the light transmittance of the film because of its relatively homogeneous distribution in the SPI matrix. The UV/Vis spectra of SPI-based films are shown in Fig. 3b, where it was clear that the transparency of SPI/PVA/PTGE/HNTs film was lower than both neat SPI film and SPI/PTGE film but higher than both SPI/PVA film and SPI/HNTs film. These differences were due to the light-scattering caused by the nanofillers^39^.

### Morphology of SPI-based Films

**Figure 4:**
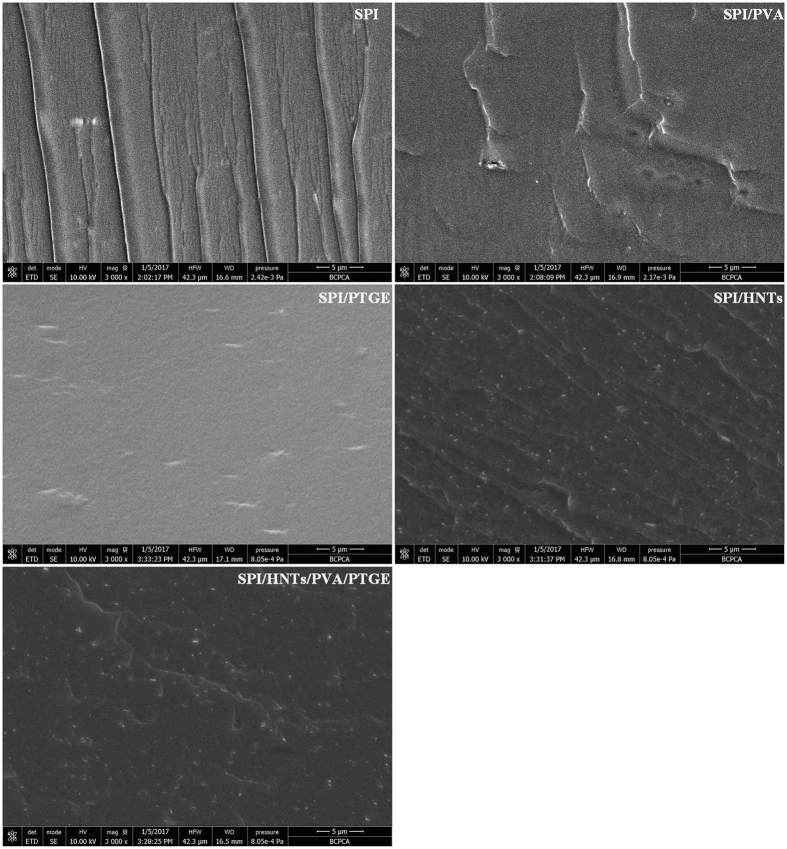
Cross-sectional SEM photographs of SPI-based films: SPI, SPI/PVA, SPI/PTGE, SPI/HNTs, and SPI/HNTs/PVA/PTGE.

The SPI films became more rigid and TS increased significantly when PVA, PTGE, and HNTs were incorporated. The cross-sectional SEM morphologies of the films are shown in Fig. 4. A relatively uneven fracture surfaces on the unmodified SPI film were observed and the river pattern indicated the cleavage fracture in SPI, which exhibited the lowest TS (2.25 MPa), while the incorporation of PVA created smoother and relatively more homogeneous cross-sections most likely due to the hydrogen bonding between SPI and PVA. More continuous and compact cross-sectional images appeared in the SPI/PTGE film due to the formation of crosslinked network structure between SPI and PTGE. These results were in agreement with our previous tests^5^. SEM images of the SPI/HNTs film indicated that HNTs were uniformly dispersed in the SPI matrix and the well-organized structure resulted in good adhesion between the fillers and matrix. The cross-sectional surface of the SPI/HNTs/PVA/PTGE film showed various and relatively uneven with several light and spotted points, which revealed synergistic effects of SPI, HNTs, PVA, and PTGE, leading to the highest TS value of the modified SPI films.

### Water Resistance of SPI-based Films

The WA and MC of unmodified and modified SPI films are summarized in Table 2. Compared to the unmodified SPI film, WA of the SPI/PVA film increased by 5.84%, which was possibly caused by the hydrogen bonding between SPI and PVA. However, WA of SPI/PTGE and SPI/HNTs films was decreased by 5.2% and 14.16%, respectively. The WA reduction of the SPI/PTGE film attributed to the formation of a crosslinked network structure between PTGE and SPI. In the SPI/HNTs film, the decrease in WA and MC might be caused by the addition of nanoparticles, resulting in increasing film tortuosity and leading to a slower diffusion of water molecules through the film matrix^40^,^41^,^42^,^43^. As indicated above, WA and MC of the SPI/HNTs/PVA/PTGE film were lower than the unmodified SPI film, but higher than the SPI/HNTs film. The observed results can be attributed to the synergistic effects of the blocking role of HNTs, the hydrophilic properties of PVA and the crosslinking agent of PTGE.

### Mechanical properties of SPI-based Films

The key parameters and mechanical properties (TS and EB) of the films as packaging materials are listed in Table 3. With the incorporation of PVA, TS of the SPI/PVA film was improved by 62.67% due to the strong hydrogen bonds formation among abundant -OH groups on SPI and PVA molecules. TS of the SPI/PTGE film was further increased by 157.33% after introducing the crosslinker PTGE. EB gradually decreased from 141.28% to 102.90% by the formation of the crosslinked network between SPI and PTGE^5^. The effects of HNTs on TS and EB of the composite film were then investigated. TS of the resultant SPI/HNTs film was increased slightly and its EB value was decreased significantly compared with the unmodified SPI film. These results were consistent with previous studies on particle-filled polymer systems, showing that the strength and toughness of materials had a fluctuating relationship^44^. This modification was made in attempt to increase the strength resulted from the physical crosslinkage. TS of the SPI/HNTs/PVA/PTGE nanocomposites film was increased by 329.33%, reaching 9.66 MPa, compared to the unmodified SPI film, which was due to the synergistic effects of SPI, HNTs, PVA and PTGE, attributing to physical and chemical crosslinking between the matrix and the filler^45^,^46^,^47^,^48^.

### Thermo-gravimetric Analysis of SPI-based Films

**Figure 5:**
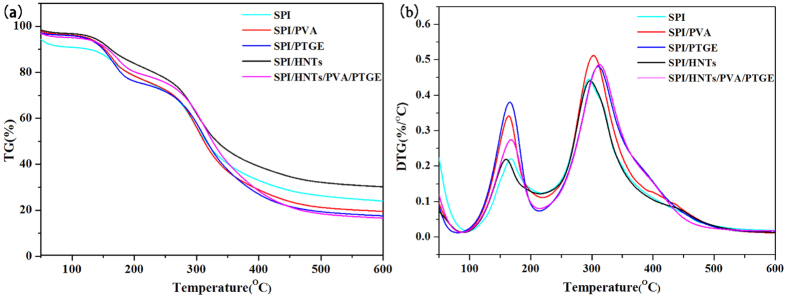
TGA (a) and DTG (b) curves of SPI-based films: SPI, SPI/PVA, SPI/PTGE, SPI/HNTs, and SPI/HNTs/PVA/PTGE.

Figure 5 shows the thermo-gravimetric analysis (Fig. 5a) and derivative thermo-gravimetric (Fig. 5b) curves of the various SPI-based films. The related degradation temperature results are summarized in Table 4. As shown in Fig. 5a, it was observed the first stage of weight loss from 30 °C to 130 °C corresponding to the evaporation of adsorbed water. The second stage of weight loss occurred from 130 °C to 270 °C, which was resulted from the evaporation of glycerol from SPI matrix. The third stage of weight loss occurred in the range from 270 °C to 450 °C because of the thermal degradation of proteins. All polymer chains decomposed or carbonized when the temperature was over 450 °C. In the second stage, the SPI/PTGE film degraded faster (greater weight loss) than that of the control, which explained the diminished toughness of the composite films due to the reduced EB^49^ (Table 4). The degradation curves of SPI-based nanocomposites showed that evident shifts occurred in the maximum degradation temperature with the presence of HNTs (Fig. 5b). The maximum temperatures (Table 4) of SPI/HNTs and SPI/HNTs/PVA/PTGE films were 297.01 °C and 312.15 °C, respectively, which were higher than the control one (295.37 °C). It was suggested that the thermal stability of SPI/HNTs and SPI/HNTs/PVA/PTGE films was enhanced compared with that of the control one, resulting in the effective delay in mass transport during the thermal decomposition via the addition of HNTs^26^,^50^. In addition, the TGA curves of the SPI/HNTs/PVA/PTGE film shifted slightly to the higher temperature, thereby indicating the increase in thermal stability, which could be reasoned by the synergism effects among SPI, HNTs, PVA, and PTGE.

## Conclusions

In this study, a series of SPI-based films were prepared via the casting method. The results showed that, with the incorporation of HNTs, PVA and PTGE, the mechanical response and thermal properties of the SPI matrix were improved without affecting its transparency, attesting to the good interfacial adhesion between the filler and matrix. Furthermore, the decrease in WA and increase in water barrier properties of SPI matrix were noticed because of the simultaneous presence of HNTs, PVA and PTGE. These findings imply that the SPI/HNTs/PVA/PTGE nanocomposite film can be a candidate as a “green” and transparent material to replace non-renewable films for the packaging applications in the future.

## Materials and Methods

### Materials

SPI with a protein content of 95% was purchased from Yuwang Ecological Food Industry Co., Ltd. (Shandong Province, China). HNTs were obtained from Lingshou County, Hebei Province, China. Industrial-grade PTGE was purchased from Chuzhou Huisheng Electronic Material Co., Ltd. (Anhui, China). Poly-vinyl alcohol (PVA) powder was purchased from Xiya Reagent Co., Ltd. Other analytical-grade chemical reactants were purchased from Beijing Chemical Reagents Co., Ltd. (China).

### Preparation of HNTs/PVA nanocomposites

The PVA solution (10%/(w/w)) and HNTs suspensions of 3 wt% concentrations based on SPI weight were firstly prepared. The eventual percolation concentration (3wt% HNTs) were described in Supporting Information (Table SI) in detail. HNT dispersion was added to the PVA solution and the mixture was continuously stirred for 1 h at room temperature and then was sonicated through a probe sonicator for 15 min. The obtained HNTs/PVA dispersion was applied for composite preparation.

### Preparation of SPI-based Films

**Figure 6:**
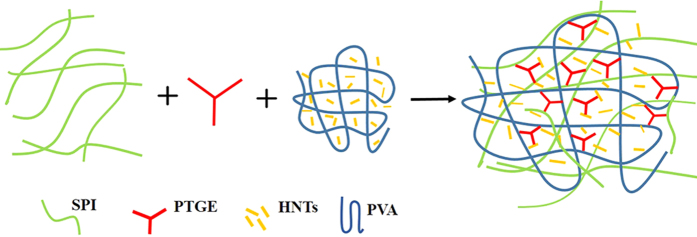
The forming mechanism of the composite film.

The nanocomposites films were prepared through the casting method^51^. Firstly, the SPI solution with varying glycerol content was adjusted to the pH value of 9.0 with NaOH solution under constant stirring for 30 min and subsequently heated to 85 °C for 30 min. Next, modifiers were added to the SPI solution and stirred uniformly. Air bubbles were removed by dropping the tributyl phosphate and ultrasound-treatment. The film-forming solutions (45 g) were cast onto plastic dishes, then dried at 45 °C for 24 h in a vacuum drying oven. All films stood at room temperature for 48 h after peeled off from the plates and stored for future use. The formulated SPI and modified SPI films are summarized in Table 5. The forming process of the composite films is presented in Fig. 6.

## Characterization

### Attenuated Total Reflectance-Fourier transformed Infrared Spectroscopy

The ATR-FTIR spectra test was carried out on a Nicolet 6700 spectrometer (Thermo Scientific, USA) in the range of 4000–650 cm^−1^ equipped with an attenuated total reflectance (ATR) accessory and conducted a total of thirty-two scans with a resolution of 4 cm^−1^ for each sample.

### X-ray Diffraction

The XRD was performed on a D8 advance diffractometer (Bruker, Germany) equipped with a Cu Kα radiation source. The diffraction data were collected from 2θ values ranging from 5° to 60° at 2° min^−1^ at 40.0 kV and 30.0 mA. The relative crystallinity index was calculated directly by the measurement instrument with Eq. (1):


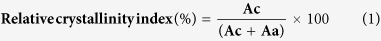


where Ac is the area of the crystalline region and Aa is the area of the amorphous region. Three replicates were carried out for each composition.

### Opacity

Opacity was measured on a UV-Vis spectrophotometer (TU-1901, Beijing Purkinje General, Beijing, China). Each film was cut to a rectangle of 2.5 cm × 1 cm and opacity was determined as the area under the absorbance curve in the visible spectrum (λ = 400–800 nm). The area was normalized and sorted by the thickness of the film.

### Scanning Electron Microscope

The cross sections morphology of different SPI films were observed with a QUANTA FEG 650 instrument (FEI, OR, USA) at an acceleration voltage of 10 kV.

### Moisture Content

Five replicates of each sample were tested to determine their MC values as described in previous studies^51^,^52^. Samples (2 cm × 2 cm) were equilibrated, weighed, and marked as m_1_, and then subsequently dried in an air-circulating oven to a constant weight (m_2_). MC was calculated as follows:


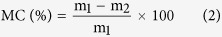


where m_1_ is the initial weight of the sample and m_2_ is the dried mass.

### Water Absorption

The WA test samples were conditioned in desiccators (0% relative humidity) with P_2_O_5_ desiccant and the initial weight (m_3_) was recorded. The water intake of the film was measured by conditioning the sample in the desiccator with distilled water to provide 92% relative humidity at room temperature (25 ± 2 °C). The films were removed after a specific period time and weighed using an electronic balance (marked m_4_). Five replicates were tested for each composition. WA was calculated as follows:


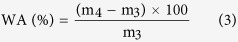


where m_3_ and m_4_ are the mass of the film before and after conditioning^5^.

### Mechanical Property and Coating Thickness

The mechanical properties of the SPI-based films were determined with a tensile testing machine (WDW3020, China) according to ISO527-3:1995(E)^53^. Each sample (10 × 80 mm^2^) was tested with a speed of 10 mm min^−1^ at room temperature. The film thickness was measured with a digimatic micrometer. The TS and EB values were the average of five replicates. All the reported values were the averages of five replicates.

### Thermal Behaviour

The thermal stabilities of the films were characterized by TGA. The non-isothermal degradation of the SPI films was performed on a Q50 TGA device (TA Instruments, USA) from room temperature to 600 °C at a heating rate of 10 °C min^−1^ under a nitrogen atmosphere (100 mL min^−1^). The samples were conditioned in desiccators (0% relative humidity) with P_2_O_5_ desiccant at room temperature until they reached a constant weight.

## Additional Information

**How to cite this article:** Liu, X. *et al*. Development of Eco-friendly Soy Protein Isolate Films with High Mechanical Properties through HNTs, PVA, and PTGE Synergism Effect. *Sci. Rep.* **7**, 44289; doi: 10.1038/srep44289 (2017).

**Publisher's note:** Springer Nature remains neutral with regard to jurisdictional claims in published maps and institutional affiliations.

## Supplementary Material

Supplementary Information

## Figures and Tables

**Table 1:** The relative Crystallinity parameters of SPI-based films.

Sample	HNTs	SPI	SPI/PVA	SPI/PTGE	SPI/HNTs	SPI/HNTs/PVA/PTGE
Crystallinity Degree (%)	71.8	23.1	15.1	29.8	34.4	36.3

**Table 2:** The WA and MC of different SPI-based films.

Specimen	WA (%)	MC (%)
SPI	82.25	12.84
SPI/PVA	87.35	12.28
SPI/PTGE	77.98	11.86
SPI/HNTs	70.60	11.04
SPI/HNTs/PVA/PTGE	78.06	11.50

**Table 3:** Thicknesses and mechanical properties (TS, EB) of SPI-based films.

Sample	Thickness (mm)	Tensile strength (MPa)	Elongation at break (%)
SPI	0.35	2.25	141.28
SPI/PVA	0.28	3.66	123.63
SPI/PTGE	0.28	5.79	102.90
SPI/HNTs	0.20	3.14	106.92
SPI/HNTs/PVA/PTGE	0.26	9.66	140.14

**Table 4:** Thermal properties of SPI-based films.

Sample	T_i1_ (°C)	T_max1_ (°C)	T_i2_ (°C)	T_max2_ (°C)
SPI	143.43	168.05	272.85	295.37
SPI/PVA	137.92	163.93	275.49	302.66
SPI/PTGE	137.90	183.92	277.94	310.51
SPI/HNTs	132.99	160.02	277.26	297.01
SPI/HNTs/PVA/PTGE	132.68	160.59	296.96	312.15

Notes: T_i_: Initial temperature of degradation.

T_max_: Temperature at maximum degradation rate.

**Table 5:** Different formulations of the SPI-based films.

Sample	SPI (g)	Glycerol (g)	Water (g)	PVA (g)^a^	HNTs (g)	PTGE (g)
SPI	3	1.5	57	0	0	0
SPI/PVA	3	1.5	57	2.2	0	0
SPI/PTGE	3	1.5	57	0	0	0.3
SPI/HNTs	3	1.5	57	0	0.09	0
SPI/HNTs/PVA/PTGE	3	1.5	57	2.2	0.09	0.3

Notes: ^a^PVA solution (10% (w/w)) was prepared by dissolving PVA in water at 90 °C.
