# Supplementary material for: Development of Eco-friendly Soy Protein Isolate Films with High Mechanical Properties through HNTs, PVA, and PTGE Synergism Effect
Source: Sci Rep. 2017 Mar 10;7:44289. doi: 10.1038/srep44289 (PMC5345057; doi:10.1038/srep44289)
Supplement: Supplementary Information [file srep44289-s1.pdf]

# Development of Eco-friendly Soy Protein Isolate Films with High Mechanical Properties through HNTs, PVA, and PTGE Synergism Effect

**Xiaorong Liu[1], Ruyuan Song[1], Wei Zhang[1], Chusheng Qi[1], Shifeng Zhang[1,*] & Jianzhang Li[1,*]**

## Supplementary Information

Table S1

Table S1. The concentration effect of the mechanical properties

| Sample | Thickness (mm) | Tensile strength (MPa) | Elongation at break(%) |
|---|---|---|---|
| SPI | 0.35 | 2.25 | 141.28 |
| SPI/1%HNTs | 0.25 | 2.63 | 101.13 |
| SPI/3%HNTs | 0.20 | 3.14 | 106.92 |
| SPI/5%HNTs | 0.23 | 3.17 | 103.44 |
